# 

**Published:** 1988-02

**Authors:** 


W1 BR323

V.103 NO.1 1988

C.01---------SEQ: B33740000

TI: BRISTOL MEDICO-CHIRURGICAL

JOURNAL

### BRISTOL MEDICO-CHIRURGICAL JOURNAL

VOLUME 103(i) FEBRUARY 1988

NATIONAL
LIBRARY
AUG 22 1988
OF
MEDICINE

Medical Education and Health Care, is there a conflict?
Crohn's Disease—a misnomer?
Harvey Cushing and the posterior pituitary.
Computer Tomography of the internal auditory canals.
A Urine Flow Clinic.
Correspondents Column on GERBIL, Cornish 'Firsts' etc.
Correspondence on AIDS, etc.
Reports from South West Surgeons.

THE MEDICAL JOURNAL OF THE SOUTH WEST

